# An Internet-Based Intervention Augmented With a Diet and Physical Activity Consultation to Decrease the Risk of Dementia in At-Risk Adults in a Primary Care Setting: Pragmatic Randomized Controlled Trial

**DOI:** 10.2196/19431

**Published:** 2020-09-24

**Authors:** Kaarin J Anstey, Nicolas Cherbuin, Sarang Kim, Mitchell McMaster, Catherine D'Este, Nicola Lautenschlager, George Rebok, Ian McRae, Susan J Torres, Kay L Cox, Constance Dimity Pond

**Affiliations:** 1 School of Psychology University of New South Wales Sydney Australia; 2 Neuroscience Research Australia Sydney Australia; 3 Centre for Research on Ageing, Health and Wellbeing Australian National University Canberra Australia; 4 Wicking Dementia Resaerch & Education Centre University of Tasmania Hobart Australia; 5 National Centre for Epidemiology and Public Health Australian National University Canberra Australia; 6 School of Medicine and Public Health University of Newcastle Newcastle Australia; 7 Academic Unit for Psychiatry of Old Age University of Melbourne Melbourne Australia; 8 Johns Hopkins Centre on Aging and Health Johns Hopkins University Baltimore, MD United States; 9 Institute for Physical Activity and Nutrition School of Exercise and Nutrition Sciences Deakin University Melbourne Australia; 10 Medical School University of Western Australia Perth Australia; 11 Department of General Practice University of Newcastle Newcastle Australia

**Keywords:** risk reduction behavior, dementia prevention & control, primary prevention, pragmatic clinical trial, prevention, primary care

## Abstract

**Background:**

There is a need to develop interventions to reduce the risk of dementia in the community by addressing lifestyle factors and chronic diseases over the adult life course.

**Objective:**

This study aims to evaluate a multidomain dementia risk reduction intervention, Body Brain Life in General Practice (BBL-GP), targeting at-risk adults in primary care.

**Methods:**

A pragmatic, parallel, three-arm randomized trial involving 125 adults aged 18 years or older (86/125, 68.8% female) with a BMI of ≥25 kg/m^2^ or a chronic health condition recruited from general practices was conducted. The arms included (1) BBL-GP, a web-based intervention augmented with an in-person diet and physical activity consultation; (2) a single clinician–led group, Lifestyle Modification Program (LMP); and (3) a web-based control. The primary outcome was the Australian National University Alzheimer Disease Risk Index Short Form (ANU-ADRI-SF).

**Results:**

Baseline assessments were conducted on 128 participants. A total of 125 participants were randomized to 3 groups (BBL-GP=42, LMP=41, and control=42). At immediate, week 18, week 36, and week 62 follow-ups, the completion rates were 43% (18/42), 57% (24/42), 48% (20/42), and 48% (20/42), respectively, for the BBL-GP group; 71% (29/41), 68% (28/41), 68% (28/41), and 51% (21/41), respectively, for the LMP group; and 62% (26/42), 69% (29/42), 60% (25/42), and 60% (25/42), respectively, for the control group. The primary outcome of the ANU-ADRI-SF score was lower for the BBL-GP group than the control group at all follow-ups. These comparisons were all significant at the 5% level for estimates adjusted for baseline differences (immediate: difference in means −3.86, 95% CI −6.81 to −0.90, *P*=.01; week 18: difference in means −4.05, 95% CI −6.81 to −1.28, *P*<.001; week 36: difference in means −4.99, 95% CI −8.04 to −1.94, *P*<.001; and week 62: difference in means −4.62, 95% CI −7.62 to −1.62, *P*<.001).

**Conclusions:**

A web-based multidomain dementia risk reduction program augmented with allied health consultations administered within the general practice context can reduce dementia risk exposure for at least 15 months. This study was limited by a small sample size, and replication on a larger sample with longer follow-up will strengthen the results.

**Trial Registration:**

Australian clinical trials registration number (ACTRN): 12616000868482; https://anzctr.org.au/ACTRN12616000868482.aspx.

## Introduction

### Background

In 2015, it was estimated that approximately 47 million people were living with dementia, and this number is expected to reach 131 million by 2050 [1]. It is estimated that a 10%-25% reduction in 7 key risk factors could prevent 1.1 to 3.0 million cases of dementia worldwide [2,3]. Furthermore, the risk factors for dementia are shared with risk factors for other chronic diseases [4]. Interventions that reduce the risk of dementia are therefore likely to also reduce the risk of other chronic diseases and promote healthy aging. In relation to cognition, prevention and delay of cognitive impairment will also benefit individuals and reduce health service usage [5-7].

To afford a greater chance of producing detectable changes during study time frames, the dementia research community has increasingly focused on multidomain interventions that address multiple risk factors simultaneously [8]. Alzheimer disease (AD) is the most common cause of dementia. Reviews of cohort studies have found that a similar set of risk factors is associated with AD and all-cause dementia [3]. Among individuals with high risk factor burden, cognitive decline can be reduced (and possibly reversed) by cardiovascular risk reduction, by increasing activities that stimulate and protect the brain, including cognitive [9], social [10], and physical activity [11], and by an appropriate diet [12,13]. Dementia and cardiovascular diseases share cardiometabolic and lifestyle risk factors [4]. Both cardiovascular disease and dementia risk reduction can be achieved by smoking cessation; increasing physical activity; adopting a healthy diet; reducing abnormally high blood pressure and cholesterol in midlife; and managing major depression, overweight or obesity in midlife, and diabetes if present [14]. Altogether, the literature supports the view that multidomain interventions aimed at reducing cardiometabolic risk and promoting behaviors protective against dementia will contribute to preventing cognitive decline, reducing the overall risk of dementia, and lowering depressive symptoms [15,16].

To bring about risk reduction and implement current guidelines on dementia risk reduction [17], there needs to be long-lasting behavioral changes in multiple areas. Moreover, strategies are required to identify adults with risk factors for dementia and to encourage them to make appropriate lifestyle changes. Achieving this requires developing pragmatic interventions that could be implemented in existing health or community settings [18,19] and using techniques such as goal setting, decreasing barriers to change, improving self-monitoring, increasing access to information, and maintaining motivation [20,21]. Therefore, this randomized controlled trial (RCT) investigated whether lifestyle management programs that offer not only health-promoting information but also practical behavior change techniques that can be implemented in daily life can reduce dementia risk.

### Objectives

Assessment of cardiovascular risk factors is common in primary care, as is lifestyle advice. General practice is therefore a setting where the need for dementia risk reduction interventions may be identified and where interventions may be prescribed [22]. Studies have been conducted in a primary care setting with older adults to address the management of cardiovascular risk factors [23,24] to reduce the risk of dementia. However, the current program is the first of its kind to provide interventions to adults (aged ≥18 years) in the primary care setting, addressing both cardiovascular and lifestyle risk factors of dementia. Specifically, the objectives of this study are to determine, in community-dwelling adults who are overweight or have chronic health conditions, the effectiveness of (1) a web-based multidomain dementia risk reduction intervention (Body Brain Life in General Practice [BBL-GP]) developed by the authors, in comparison to (2) a single clinician–led group, Lifestyle Modification Program (LMP), developed by a general practice cooperative, and (3) a web-based active control condition developed by the authors.

## Methods

### Trial Design

The trial protocol for the BBL-GP study is published elsewhere [25]. Briefly, it is a three-arm, pragmatic, single-blind RCT to reduce the risk of cognitive decline in at-risk individuals attending a general practice. The study was conducted within the National Health Co-op (NHC), a bulk billing general practice (ie, patients’ general practitioner [GP] fees are fully funded by a universal health cover Medicare scheme) in Canberra, with 8 clinics drawn from low- and middle-income areas. Canberra has a cold climate and a slightly higher than average level of education compared with the national average in Australia [26]. It also has a lower rate of bulk billing primary care services than any other state or territory in Australia [27]. The NHC had an existing program called the LMP, to which practice physicians referred patients diagnosed with or at-risk of chronic health conditions. To integrate the trial into existing referral pathways at the clinics, the referral criteria for LMP were used as the trial eligibility criteria, and this was managed by the clinic’s LMP coordinator.

### Participants

Recruitment occurred via email, posters displayed in the NHC clinics, and the NHC website. Participants who expressed interest in the study were contacted by the LMP coordinator for a screening assessment of inclusion and exclusion criteria (outlined below). After eligible volunteers provided written informed consent, they were provided log-in details for the BBL-GP study website for web-based data collection purposes; a clinic appointment was also organized. Participant recruitment was undertaken over a 12-month period from mid-July 2016 until the end of July 2017, and the final follow-ups were completed in December 2018. Appointments were booked by the NHC staff. The research team was not involved in recruitment or assessment. The Australian National University Human Research Ethics Committee approved the study.

### Eligibility Criteria

The eligibility criteria included being aged 18 years or older, being a resident in the Australian Capital Territory or surroundings, being a registered member of the NHC (which involves paying a joining fee of AUD $30 [US $21.90] and an annual fee of AUD $100 [US $73]), having home access to a computer and internet, having English fluency, having Australian permanent residency or citizenship (for universal health cover eligibility), being the sole member of a household taking part in the study, and having a chronic health condition that would make the participant eligible for the NHC lifestyle management program (eg, hypertension, heart disease, type 2 diabetes, osteoporosis, polycystic ovary syndrome, kidney or liver disease) or being overweight or obese (BMI ≥25 kg/m^2^). All participants aged older than 60 years were required to score within the nonimpaired range on the Mini-Mental State Examination (MMSE; ≥25) [28] to be enrolled in the study.

Exclusion criteria included significant and unstable medical and psychiatric conditions precluding participation, sensory deficits, or mobility limitations that would prevent completion of the interventions, cognitive impairment (including AD or dementia), pregnancy, and previous participation in the LMP.

### Interventions

The BBL-GP intervention is a 12-week program that includes (1) 8 web-based electronic learning modules on dementia literacy, risk factors, physical activity, nutrition, health condition management, cognitive activity, social activity, and mood and (2) tailored, face-to-face physical activity and nutrition sessions. BBL-GP participants were required to complete all 8 modules. The dementia literacy and risk factor modules were standardized across participants and were released at the rate of one per week after completion of the prior module, with the same timing for all participants. For the remaining modules, the content was tailored to participants’ individual risk profiles as indicated by the baseline Australian National University Alzheimer Disease Risk Index Short Form (ANU-ADRI-SF; described below). The tailoring algorithms are included in Multimedia Appendix 1. These modules were also released at the rate of one per week, which was the same for all participants. The same exercise physiologist and 3 dietitians conducted all face-to-face sessions for the BBL-GP participants. The dietitians and exercise physiologist were all staff members within the NHC, but their services for this project were funded by a research grant. Participants at baseline who had unintentional weight loss or weight gain (±5 kg) or scored low on the Australian Recommended Food Score were seen by one of the dietitians (1-hour face-to-face) [25] and reviewed via phone at weeks 4, 12, and 20. Each participant received a single exercise physiology session that involved an evaluation of their current exercise level, fitness, and any preexisting health conditions and the design of a personal exercise program. Although follow-up via phone was planned, this was not conducted. The content of the dietitian’s session was dietary education and advice to assist the participant in adapting their diet to a healthy diet in areas that were identified as unhealthy in the dietary questionnaire. Diet and exercise physiologist sessions varied depending on the participants’ clinical needs and baseline measures. All sessions related to the BBL-GP were provided free of charge to the participants.

The LMP intervention was a face-to-face, practice-based, 6-week program provided by the NHC to its members for free. It included group sessions providing generic information on basic nutrition, meal planning, physical activity, health conditions, motivation and goals, medications, and sleep. All LMP sessions were conducted by an NHC clinical staff member using existing clinic resources. Each LMP group session involved up to 20 participants, and relevant sessions were conducted by one of the 3 dietitians and an exercise physiologist as for the BBL-GP group.

The 12-week active control arm involved weekly emails sent to participants that included links to information regarding lifestyle risk factors and disease management. At the end of the intervention, participants in the active control group received a 1-hour, face-to-face, group-based risk reduction workshop that provided the information contained in the BBL-GP intervention. This was held at the Australian National University and involved 1 to 6 participants and was delivered by KA with assistance from SK and MM.

Participants from all groups were sent a standard email when they were due for their follow-up appointment (18, 32, and 62 weeks). They were asked to complete their web-based assessments that were located on the BBL website, plus a diet quiz for which links were provided. If the web-based follow-up was not completed after 1 week, participants received a follow-up phone call from the research team with additional phone calls to request completion of missing sections of the assessments. The NHC receptionist arranged follow-up appointments for clinical assessments. The assessment was treated as missing after 3 follow-up phone calls. At the completion of the trial, participants were invited to complete a feedback questionnaire on the web, which included structured questions and one open-ended comment.

### Randomization and Blinding

Following completion of the baseline assessment, consenting participants were randomized to one of the 3 intervention groups in a 1:1:1 ratio, stratified by sex and age group (18-49 vs ≥50 years) using permuted blocks of 6. Randomization was performed using the Sealed Envelope software [29] by a researcher not involved in the study, and the allocation sequence was provided to the project manager, who was also not involved in conducting assessments. The project manager then assigned the participant to their intervention group according to the schedule and notified the participant of their group allocation via email. Participants were not blinded to the group allocation.

Study conditions were presented as 3 alternative lifestyle interventions in recruitment materials. Participants were not informed of the intervention of interest to the researchers.

Web-based research data were stored on a server at the Australian National University, which was compliant with Australian data protection laws.

### Outcomes

Web-based surveys and face-to-face visits to the NHC were conducted for the baseline evaluation and for the 18-, 36-, and 62-week follow-ups. Immediate follow-up (at the end of the formal program) was conducted on the web at week 7 for LMP and week 13 for the BBL-GP and control groups. These times were chosen to evaluate the immediate effects and the long-term effects of the intervention.

The web-based questionnaire included the primary outcome measure, the ANU-ADRI-SF [30]. The ANU-ADRI-SF is a shortened version of the ANU-ADRI [31]. Intraclass correlation coefficients assessing the agreement of individual components on the original and short-form ANU-ADRI were high (0.77-0.99). The ANU-ADRI has been externally validated in 3 cohort studies to predict dementia [32] and on a fourth cohort to predict mild cognitive impairment (MCI) [33]. The ANU-ADRI-SF questionnaire included secondary outcome measures of self-reported physical activity using the short version of the International Physical Activity Questionnaire (IPAQ) [34]. Additional questionnaires were the Pittsburgh Sleep Quality Index (PSQI) [35], the 12-item Short-Form Health Survey (SF-12) [36], and the Australian Recommended Food Score (ARFS) [37]. The web-based baseline assessment also included the Multidimensional Health Questionnaire [38] and the Adult Pre-exercise Screening System (APSS) [39] to identify individuals with acute or high-risk conditions or those who may be at higher risk of adverse events during exercise. The results of the APSS were provided to the exercise physiologist before the one-to-one exercise session with participants in the BBL-GP group.

Following completion of the web-based surveys, participants underwent a series of assessments at one of the 5 NHC clinics. Participants were assessed on sociodemographic characteristics, anthropometric measures, smoking status, alcohol consumption, blood pressure, cholesterol, high-density lipoprotein, blood glucose, social history (marital status and living arrangements), recreational activities, medical history, medications, and family history. From the information collected, the Framingham cardiovascular disease (CVD) risk score and the Australian Type 2 Diabetes Risk Assessment Tool risk scores were calculated. Body fat composition was also measured at the clinic using a bioelectrical impedance analysis, accounting for age, sex, height, and weight. Cognitive measures included the Trail Making Test A and B [40] and the Symbol Digit Modalities Test [41], which were completed via the BBL website on a desktop computer while participants were at the clinic in the presence of a clinic nurse. The assessments were conducted entirely by practice nurses. The MMSE was administered to individuals aged 60 years or older at baseline (see the Eligibility Criteria section). Objective physical activity was measured using the duration of moderate-to-vigorous physical activity (MVPA) via an ActiGraph monitor (GT9X Link) worn on the wrist for 7 days following the participants’ clinic visit. MVPA was calculated as a continuous measure of activity registering 3 or more metabolic equivalents for 10 min or longer on the activity monitor. Self-reported physical activity was measured as part of the ANU-ADRI-SF using IPAQ categories for high, moderate, and low levels of physical activity, and relevant risk scores were assigned as part of ANU-ADRI.

### Compliance

Compliance was measured by completion of web-based modules, following recommendations provided by the dietitian and exercise physiologist, or attendance at LMP group sessions, where relevant [25].

### Harms and Other Adverse Events

Study team members monitored and managed any risks throughout the trial, including data handling and website access.

### Participant Experience and Open-Text Feedback

At the conclusion of study participation, each participant completed a feedback questionnaire requiring them to rate the following aspects on a 5-point scale: overall study experience, perceived relevance of the intervention materials, level of interest in materials, and perceived repetition of intervention materials. Participants also rated whether participation was worthwhile and their perceived effectiveness of the group to which they were randomized.

### Sample Size

We planned to recruit 240 participants to detect a difference in continuous outcomes between groups of SD of 0.5 based on ANU-ADRI (medium effect, based on a previous BBL project [42]), assuming 80% power and a two-tailed 5% significance level and allowing for 33% attrition over time (based on the previous LMP programs in the NHC).

### Statistical Methods

For each outcome, analysis involved the generation of regression models (linear regression for continuous outcomes: primary outcome of ANU-ADRI and secondary outcomes of standardized cognition score, MVPA per week, ARFS, and PSQI; negative binomial regression for highly right-skewed continuous or count data: Center for Epidemiological Studies Depression Scale [CES-D]; and logistic regression for binary outcomes: proportion with sufficient physical activity), including age, sex, outcome at each time point, intervention group, time point, and the interaction between the intervention group and time point. Mixed models were used to adjust for the correlation of outcome within individuals over time. The interaction term allowed comparison of outcomes between intervention groups at each time point, unadjusted (primary estimates) and adjusted (secondary estimates) for baseline values, using the *lincom* command in Stata (StataCorp LLC). For continuous outcomes, the differences in means between groups (unadjusted and adjusted for baseline differences in outcome) were obtained with 95% CIs. For binary outcomes, odds ratios with 95% CIs were obtained unadjusted for baseline differences, whereas the adjusted estimates or *intervention effect* was estimated as the ratio of odds ratios for the relevant time point and baseline: that is, the odds ratio at the time point of interest divided by the odds ratio at baseline, with 95% CIs. Outcomes with 95% CIs (means for continuous outcomes and proportions for binary outcomes) were graphed for the intervention group at each time point. A sensitivity analysis adjusted for (in addition to age and sex) sufficient physical activity, standardized cognition score, ARFS (diet), SF-12 Physical Component Score, SF-12 Mental Component Score, and Framingham CVD risk score at baseline was undertaken because these variables were considered to be potentially associated with the outcomes or attrition.

The primary analysis was a complete case analysis [43] for all outcome measures. The secondary analysis involved multiple imputations to account for missing data in a full intention-to-treat analysis. Variables included in the imputation model were sociodemographic characteristics (age, sex, and years of education at baseline), intervention group, values of the outcome at each time point, and baseline values of BMI and SF-12. Imputation was undertaken for all 3 intervention groups combined, rather than separately for each group, because of the small number of observations within each group and the number of observations with missing data. A total of 50 imputation data sets were generated, and an overall estimate was obtained according to the Rules by Rubin [44].

## Results

### Baseline Characteristics

**Figure 1:**
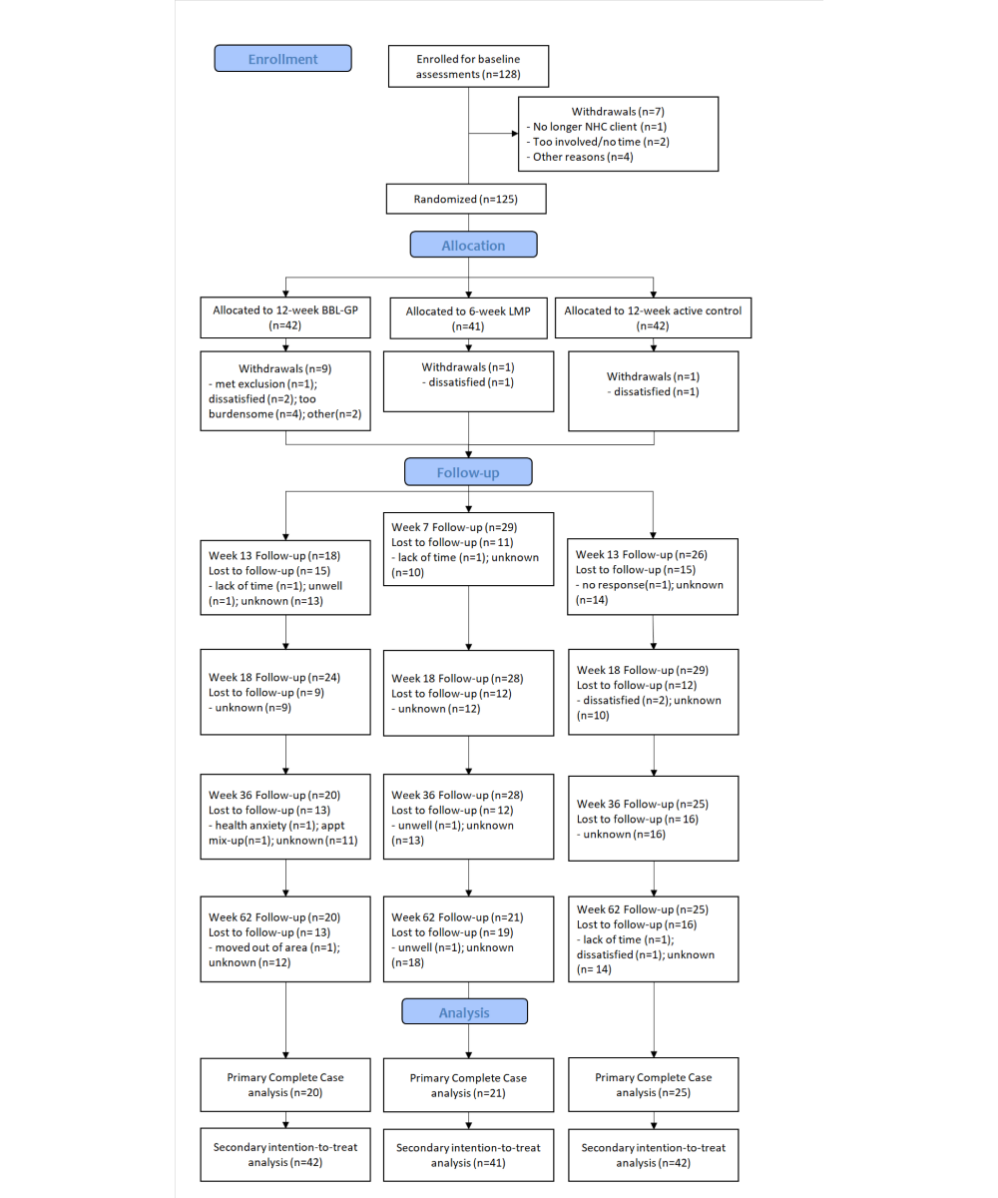
Participant flow through the trial, follow-up, and analysis. BBL-GP: Body Brain Life in General Practice; LMP: Lifestyle Modification Program; NHC: National Health Co-op.

In total, 125 patients were recruited (42 in the BBL-GP group, 41 in the LMP group, and 42 in the control group). Figure 1 shows the flow of participants through the trial and loss to follow-up for each of the 3 intervention groups. Overall, 31% (13/42) individuals in the BBL-GP group, 41% (17/41) in the LMP group, and 45% (19/42) in the control group provided data at all 5 time points. Although a smaller number of participants were recruited than planned, recruitment was stopped because of funding limitations. The planned intervention and follow-up durations did not change. A total of 66 individuals provided data at week 62 (20 in the BBL-GP group, 21 in the LMP group, and 25 in the control group). Baseline characteristics and outcomes at baseline are shown for the 3 intervention groups in Table 1. The 3 groups were generally well-matched, although the control group appeared to have a slightly lower BMI and ANU-ADRI-SF score and slightly higher scores on CES-D and the BBL-GP group had slightly more minutes of MVPA per week.

**Table 1:** Baseline characteristics by intervention group.

Variable	Body Brain Life in General Practice		Lifestyle Modification Program		Control	
	N^a^	Values, mean^b^ (SD) or n (%)	N^a^	Values, mean^b^ (SD) or n (%)	N^a^	Values, mean^b^ (SD) or n (%)
Female	42	28 (67%)	41	27 (66%)	42	31 (74%)
Age, years	42	51.14 (14.24)	41	51.41 (11.69)	42	49.95 (14.03)
Education, years	42	15.51 (4.49)	41	16.41 (4.25)	42	16.11 (4.16)
BMI, kg/m^2^	42	34.52 (7.11)	41	34.55 (6.65)	42	32.91 (7.36)
Australian National University Alzheimer Disease Risk Index Short Form	37	3.22 (7.13)	37	3.11 (7.19)	38	1.82 (6.36)
Cognition *z* score	41	−0.1 (1.01)	37	0.03 (1.06)	41	0.07 (0.94)
Total MVPA^c^ per week^d^	40	1041.78 (528.97)	36	868.31 (409.62)	37	892.51 (420.01)
Sufficient physical activity	37	19 (51%)	37	20 (54%)	38	20 (53%)
Center for Epidemiological Studies Depression Scale score	42	7 (4,15)	41	7 (3,15)	42	10 (4,14)
Diet (Australian Recommended Food Score)	42	35.86 (8.84)	38	35.92 (10.42)	40	36 (8.36)
Sleep (Pittsburgh Sleep Quality Index)	41	6.63 (3.67)	41	7.46 (4.27)	41	8.02 (4.03)
12-item Short-Form Health Survey Physical Component Score	42	45.21 (8.62)	39	47.12 (10.49)	42	44.62 (7.93)
12-item Short-Form Health Survey Mental Component Score	42	44.95 (11.26)	39	45.42 (11.74)	42	42.81 (11.10)
Diabetes risk (Australian Type 2 Diabetes Risk Assessment Tool)	40	15.88 (6.53)	38	16.92 (5.66)	40	16.3 (5.56)
Framingham cardiovascular disease risk score	38	3.5 (0,7)	35	3.0 (1,7)	34	3.0 (0,6)

^a^Numbers may not add to the total sample size because of missing values.

^b^Median (Q1 and Q3) presented for the Center for Epidemiological Studies Depression Scale and Framingham cardiovascular disease risk scores.

^c^MVPA: moderate-to-vigorous physical activity.

^d^Total minutes of MVPA per week (activity registering 3 or more metabolic equivalents for at least 10 min).

### Completion Rates and Missing Data

Multimedia Appendix 2 shows the characteristics of individuals who did (completers) and did not (noncompleters) provide data at all 5 time points. Although there were no statistically significant differences between completers and noncompleters, the sample size was small, and thus, statistical power was low for these comparisons. Some individuals did not provide information on the number of hours of each type of physical activity undertaken, resulting in missing data for both the IPAQ measure and the overall ANU-ADRI score.

### Intervention Effects

**Figure 2:**
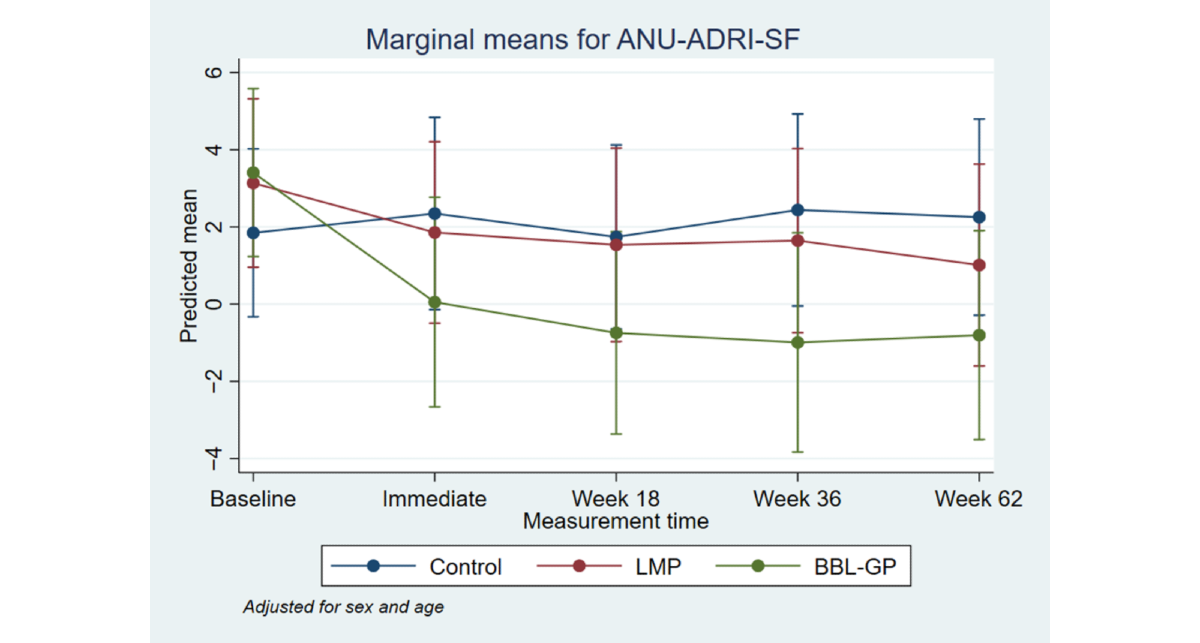
Mean Australian National University Alzheimer Disease Risk Index Short Form with 95% CI, by time point and intervention group. ANU-ADRI-SF: Australian National University Alzheimer Disease Risk Index Short Form; BBL-GP: Body Brain Life in General Practice; LMP: Lifestyle Modification Program.

**Figure 3:**
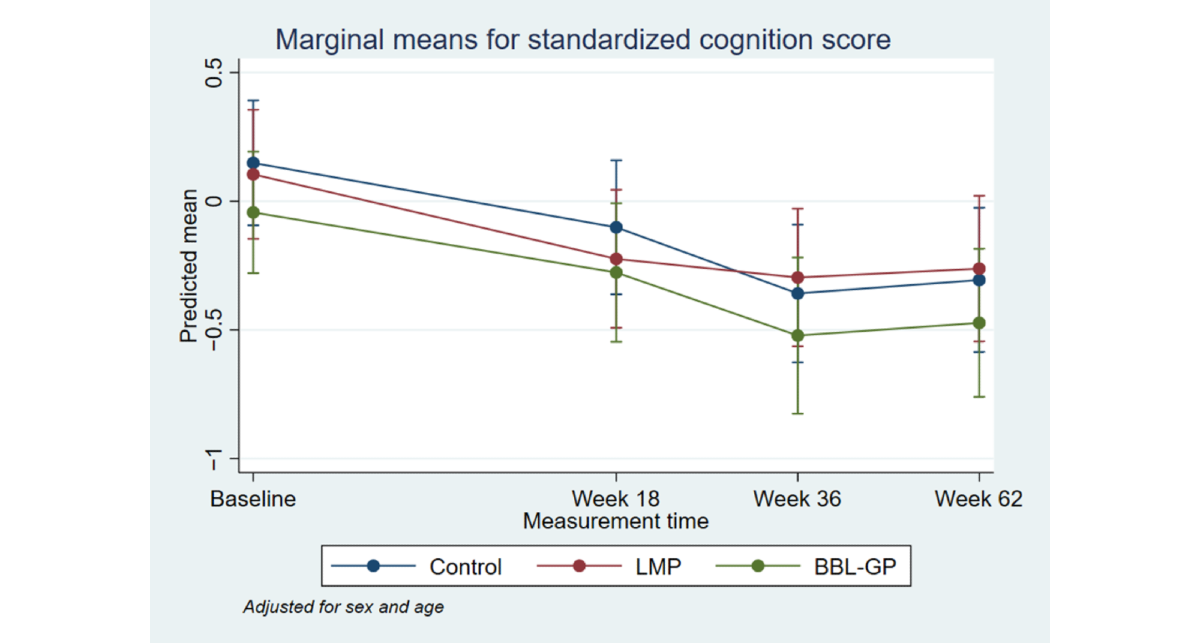
Mean standardized cognition score with 95% CI, by time point and intervention group. BBL-GP: Body Brain Life in General Practice; LMP: Lifestyle Modification Program.

**Figure 4:**
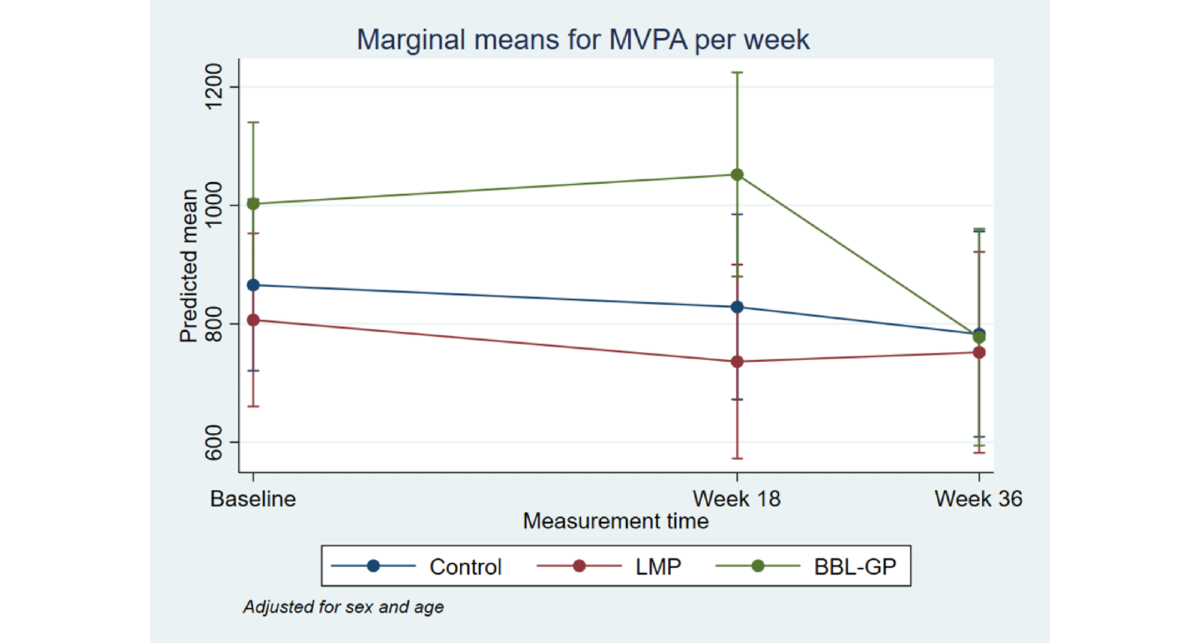
Mean moderate-to-vigorous physical activity with 95% CI, by time point and intervention group. BBL-GP: Body Brain Life in General Practice; LMP: Lifestyle Modification Program; MVPA: moderate-to-vigorous physical activity.

**Figure 5:**
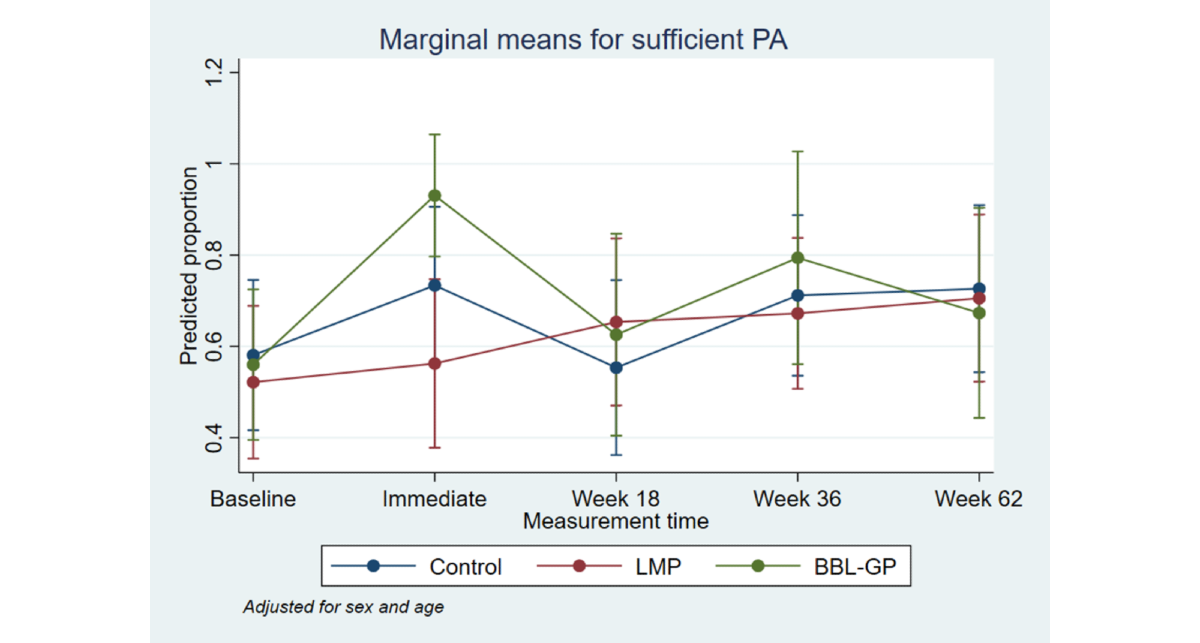
Proportion with sufficient physical activity with 95% CI, by time point and intervention group. Although the upper limit of some 95% CIs has been estimated as >1, it is not possible for a proportion to exceed 1. BBL-GP: Body Brain Life in General Practice; LMP: Lifestyle Modification Program; PA: physical activity.

**Figure 6:**
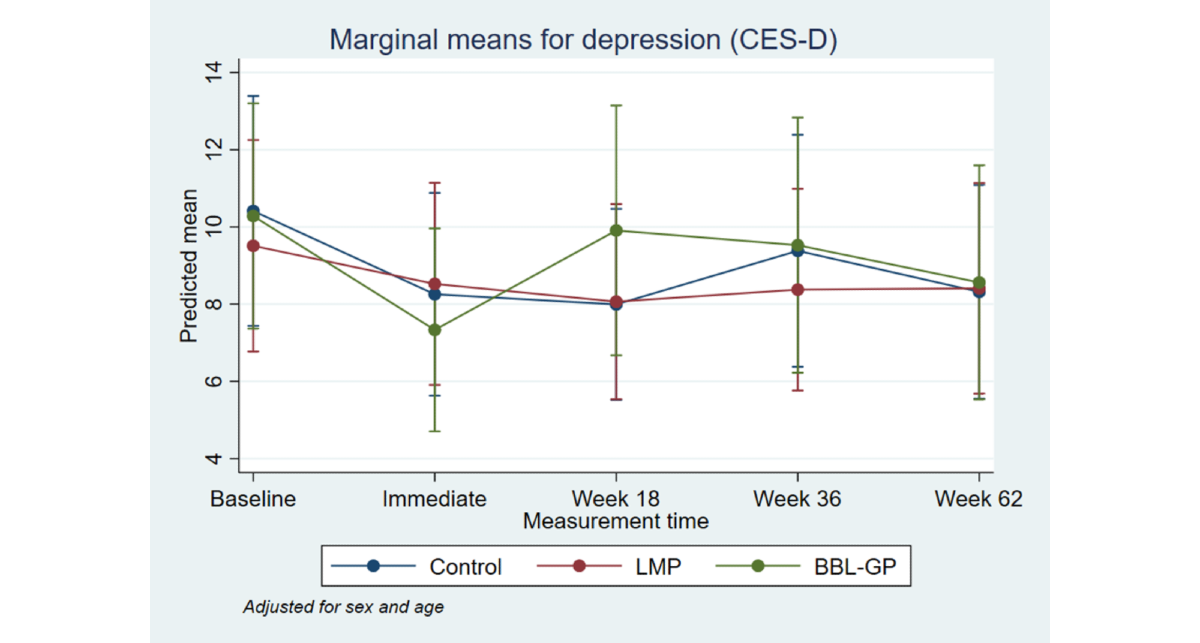
Mean Center for Epidemiological Studies Depression Scale with 95% CI, by time point and intervention group. BBL-GP: Body Brain Life in General Practice; CES-D: Center for Epidemiological Studies Depression Scale; LMP: Lifestyle Modification Program.

**Figure 7:**
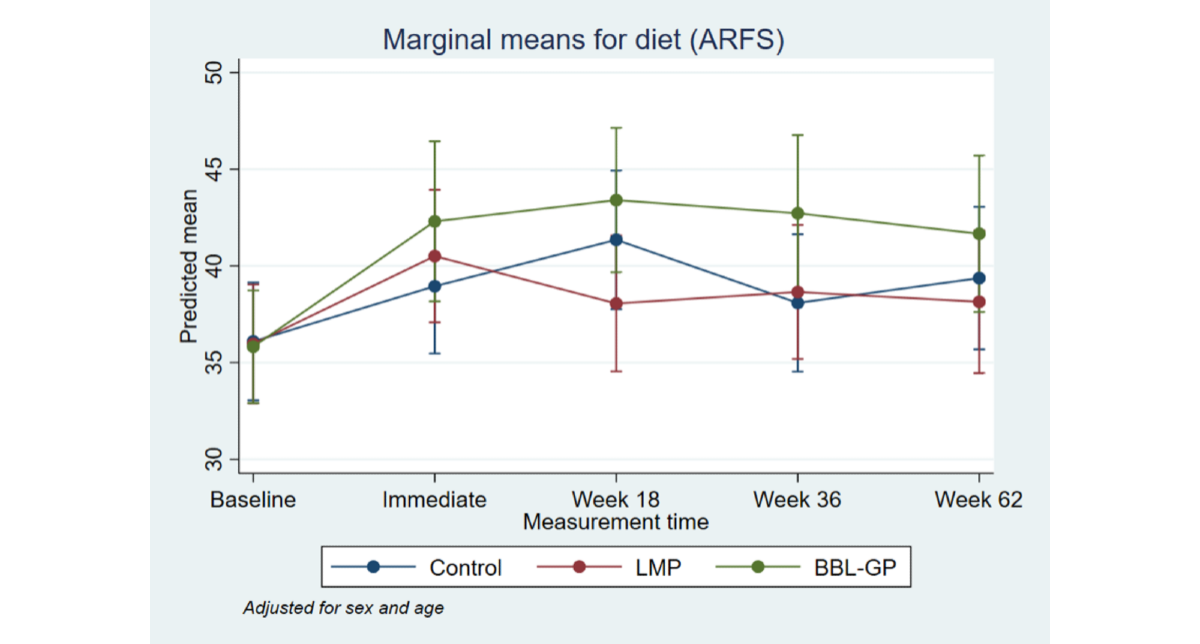
Mean Diet Score (ARFS) with 95% CI, by time point and intervention group. ARFS: Australian Recommended Food Score; BBL-GP: Body Brain Life in General Practice; LMP: Lifestyle Modification Program; PSQI: Pittsburgh Sleep Quality Index.

**Figure 8:**
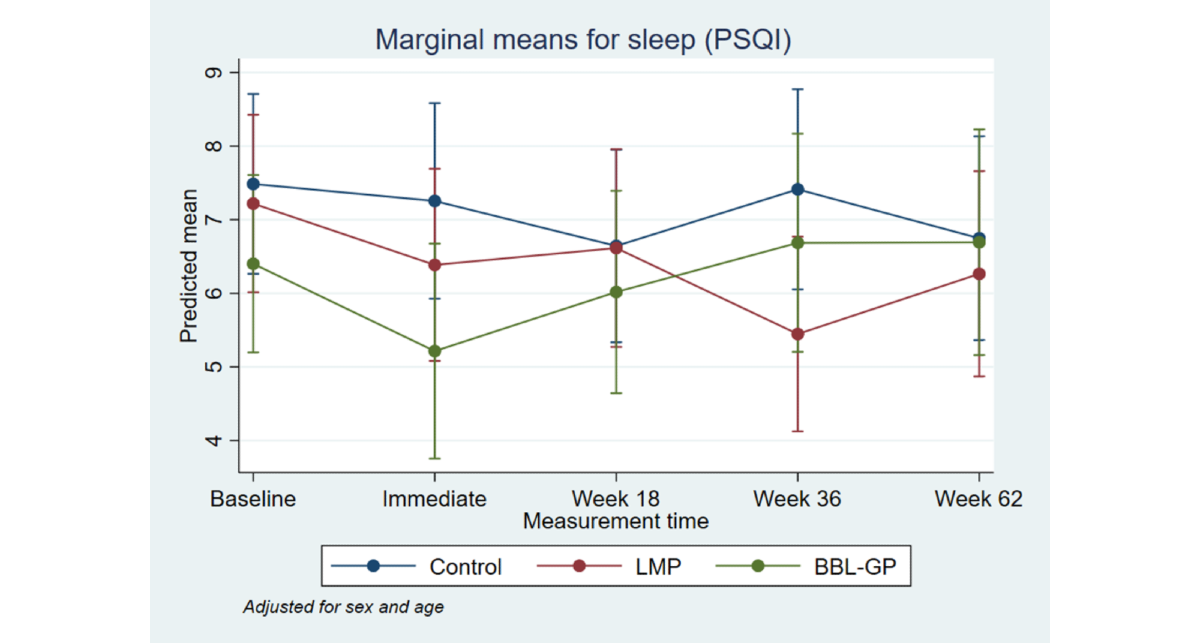
Mean Sleep Score (PSQI) with 95% CI, by time point and intervention group. BBL-GP: Body Brain Life in General Practice; LMP: Lifestyle Modification Program.

Figures 2 to 8 and Multimedia Appendix 3 show the results of the comparison of outcomes between groups at each follow-up time from the models adjusted for age and sex. The interaction terms were statistically significant for the ANU-ADRI-SF and the PSQI, indicating significant group differences over time. However, there was only a clear and consistent pattern of better outcomes over time associated with the interventions for the primary outcome, ANU-ADRI-SF. This shows that the BBL-GP intervention reduced the risk of dementia. The difference between BBL-GP and the control group was only statistically significant at the 10% level for weeks 36 and 62 for ANU-ADRI-SF, although the trend for reduced risk scores remained (immediate: difference in means −2.30, 95% CI −5.93 to 1.34, *P*=.22; week 18: difference in means −2.49, 95% CI −5.99 to 1.02, *P*=.16; week 36: difference in means −3.43, 95% CI −7.16 to 0.29, *P*=.07; week 62: difference in means −3.06, 95% CI −6.71 to 0.60, *P*=.10). After adjusting for baseline differences in outcomes, in addition to age and sex, the differences in mean ANU-ADRI-SF between the BBL-GP and control groups increased (Multimedia Appendix 4) and were statistically significant at the 5% level at each follow-up time (immediate: difference in means −3.86, 95% CI −6.81 to −0.90, *P*=.01; week 18: difference in means −4.05, 95% CI −6.81 to −1.28, *P*<.001; week 36: difference in means −4.99, 95% CI −8.04 to −1.94, *P*<.001; week 62: difference in means −4.62, 95% CI −7.62 to −1.62, *P*<.001). Although the graph of mean diet score demonstrated some indication of higher scores for BBL-GP relative to the other group, this was not statistically significant, possibly because of low power.

### Sensitivity or Secondary Analyses

Sensitivity analyses adjusted for baseline physical activity, cognition, diet, mental and physical health, and CVD risk and secondary analyses using multiple imputation produced results that were broadly consistent with the primary analyses (although it was not possible to undertake multiple imputations for all secondary outcomes because of the lack of convergence of imputation and/or analysis models).

### Compliance

Although adjustment for adherence (compliance) was originally planned [25], compliance was not considered in the analyses because of the smaller-than-anticipated number of individuals recruited and the amount of loss to follow-up.

### Harms and Other Adverse Events

No adverse events were reported. There were no privacy breaches and no major technical problems. Occasional issues with accessing the website were reported and dealt with by the study team.

### Participant Experience and Open-Text Feedback

A summary of the participant feedback on their experience of the intervention is found in Multimedia Appendix 5. A total of 21/29 (72.4%) participants who provided feedback rated their study experience as good or very good, and 20/26 (77%) felt their participation was worthwhile.

## Discussion

### Principal Findings

Our study aimed to evaluate the effectiveness of a multidomain lifestyle intervention in primary care to reduce the risk of dementia compared with a lifestyle management program that was already offered to primary care patients and an internet control condition. The significant group-by-time interaction in our main analysis showed that the multidomain BBL-GP lifestyle intervention was effective in reducing the risk of dementia for a period of at least 15 months. This builds on a growing body of research supporting the benefits of multidomain interventions that focus specifically on factors impacting brain health [8,45]. In contrast, a more generic healthy lifestyle management program that was not personalized did not result in dementia risk reduction. Population-based research has shown that participants aged 60 to 64 years with a one-point lower ANU-ADRI at baseline have an 8% lower chance of developing MCI or dementia [33,46] over a 12-year period. Our intervention achieved a lower ANU-ADRI-SF score of 4.62 (0.62 SD or equivalent to approximately 2 risk factors) at 62 weeks for the BBL-GP group relative to the control group in our analyses, which adjusted for baseline differences in the ANU-ADRI-SF. We therefore infer that maintenance of these benefits would translate into a significant shift in the risk of dementia at the population level, even if the effect size reduced over time.

### Limitations and Strengths

Our study had a number of limitations. It was underpowered because of the smaller-than-anticipated sample and the pragmatic design whereby all the assessments and follow-ups were arranged by the NHC staff who assisted with the trial in addition to their usual workloads. This limited our capacity to evaluate the secondary outcomes. The sample attrition was relatively high with regard to attendance at follow-up appointments. We hypothesize that this was partly because of the limited role of the research team in arranging appointments for participants. The study was not resourced for vigilant follow-up of participants, which differs from our previous trial where a research assistant undertook this role [47]. This study utilized the desired model of the NHC and provided a seamless patient experience, including the use of NHC staff in their usual locations conducting assessments. Future research is needed to develop mechanisms to increase participant follow-up compliance, co-designed with the general practice. In addition, more evidence is required to inform the design of interventions with regard to the number of allied health sessions and the efficacy of using web-based video to administer these sessions. A further study limitation was the relatively short length of follow-up.

The strengths of our study are that the trial was designed in partnership with a large primary care provider using the practice staff, space, and booking systems so that patients from the practice could complete the intervention within their usual settings. It was also implemented by the clinical staff without research training, which is more realistic than most research studies. It is therefore generalizable to the wider primary care setting in Australia. Our study also included 2 comparison conditions, including a lifestyle intervention that is used in clinical practice. Although it was an RCT, the study was implemented in such a way that the practice could continue the program if the BBL-GP site and allied health sessions could be supported and funded. Hence, we were able to demonstrate the benefits of a dementia-specific, multidomain lifestyle intervention. Our outcome measure, being a composite assessment, was sensitive to multidomain risk reduction.

### Implications and Conclusions

This trial has several practical implications. The approach to patient selection was straightforward and compatible with usual care. GPs only need to identify patients who have a chronic condition and are appropriate for a lifestyle intervention to select those who will also benefit from a dementia risk reduction intervention. Participants were able to participate in the web-based component of the intervention in their own time and at minimum cost. The use of specialist practitioners to deliver the physical activity and nutrition sessions meant that these were tailored to the individual’s clinical profile. This enabled broad inclusion criteria because the clinical risk was managed appropriately. Often, patients with multiple chronic conditions are excluded from trials, which reduces the ecological validity and generalizability of findings to the wider population. We conclude that dementia risk reduction in an adult population is feasible in the primary care setting and worthy of further research to establish scalable and sustainable models.

## Appendix

Multimedia Appendix 1 Algorithm for tailoring risk factor intervention modules.

Multimedia Appendix 2 Baseline characteristics by completion.

Multimedia Appendix 3 Difference in outcomes between groups at each follow-up.

Multimedia Appendix 4 Difference in outcomes between groups at each follow-up, adjusted for baseline differences.

Multimedia Appendix 5 Summary of participant feedback.

## Abbreviations

AD: Alzheimer disease
ANU-ADRI-SF: Australian National University Alzheimer Disease Risk Index Short Form
APSS: Adult Pre-exercise Screening System
ARFS: Australian Recommended Food Score
BBL-GP: Body Brain Life in General Practice
CES-D: Centre for Epidemiological Studies Depression Scale
CVD: cardiovascular disease
IPAQ: International Physical Activity Questionnaire
LMP: Lifestyle Modification Program
MCI: mild cognitive impairment
MMSE: Mini-Mental State Examination
MVPA: moderate-to-vigorous physical activity
NHC: National Health Co-op
PSQI: Pittsburgh Sleep Quality Index
RCT: randomized controlled trial
SF-12: 12-item Short-Form Health Survey

## References

[1] Prince M, Wimo A, Guerchet M, Ali G, Wu Y, Prina M (2015). World Alzheimer's Report.

[2] Livingston G, Sommerlad A, Orgeta V, Costafreda SG, Huntley J, Ames D, Ballard C, Banerjee S, Burns A, Cohen-Mansfield J, Cooper C, Fox N, Gitlin LN, Howard R, Kales HC, Larson EB, Ritchie K, Rockwood K, Sampson EL, Samus Q, Schneider LS, Selbæk G, Teri L, Mukadam N (2017). Dementia prevention, intervention, and care. Lancet.

[3] Barnes DE, Yaffe K (2011). The projected effect of risk factor reduction on Alzheimer's disease prevalence. Lancet Neurol.

[4] Peters R, Ee N, Peters J, Beckett N, Booth A, Rockwood K, Anstey KJ (2019). Common risk factors for major noncommunicable disease, a systematic overview of reviews and commentary: the implied potential for targeted risk reduction. Ther Adv Chronic Dis.

[5] O'Donoughue Jenkins L, Butterworth P, Anstey KJ (2016). A longitudinal analysis of general practitioner service use by patients with mild cognitive disorders in Australia. Dement Geriatr Cogn Disord.

[6] Griffith LE, Gruneir A, Fisher K, Panjwani D, Gandhi S, Sheng L, Gafni A, Patterson C, Markle-Reid M, Ploeg J (2016). Patterns of health service use in community living older adults with dementia and comorbid conditions: a population-based retrospective cohort study in Ontario, Canada. BMC Geriatr.

[7] Jacobs JM, Rottenberg Y, Cohen A, Stessman J (2013). Physical activity and health service utilization among older people. J Am Med Dir Assoc.

[8] Ngandu T, Lehtisalo J, Solomon A, Levälahti E, Ahtiluoto S, Antikainen R, Bäckman L, Hänninen T, Jula A, Laatikainen T, Lindström J, Mangialasche F, Paajanen T, Pajala S, Peltonen M, Rauramaa R, Stigsdotter-Neely A, Strandberg T, Tuomilehto J, Soininen H, Kivipelto M (2015). A 2 year multidomain intervention of diet, exercise, cognitive training, and vascular risk monitoring versus control to prevent cognitive decline in at-risk elderly people (FINGER): a randomised controlled trial. Lancet.

[9] Wilson RS, Scherr PA, Schneider JA, Tang Y, Bennett DA (2007). Relation of cognitive activity to risk of developing Alzheimer disease. Neurology.

[10] Seeman TE, Miller-Martinez DM, Stein Merkin S, Lachman ME, Tun PA, Karlamangla AS (2011). Histories of social engagement and adult cognition: midlife in the US study. J Gerontol B Psychol Sci Soc Sci.

[11] Chang M, Jonsson PV, Snaedal J, Bjornsson S, Saczynski JS, Aspelund T, Eiriksdottir G, Jonsdottir MK, Lopez OL, Harris TB, Gudnason V, Launer LJ (2010). The effect of midlife physical activity on cognitive function among older adults: AGES--Reykjavik study. J Gerontol A Biol Sci Med Sci.

[12] Hosking DE, Eramudugolla R, Cherbuin N, Anstey KJ (2019). MIND not Mediterranean diet related to 12-year incidence of cognitive impairment in an Australian longitudinal cohort study. Alzheimers Dement.

[13] Prince M, Albanese E, Guerchet M, Prina M (2014). World Alzheimer Report 2014: Dementia and Risk Reduction. Alzheimer's Disease International.

[14] Santos CY, Snyder PJ, Wu W, Zhang M, Echeverria A, Alber J (2017). Pathophysiologic relationship between Alzheimer's disease, cerebrovascular disease, and cardiovascular risk: a review and synthesis. Alzheimers Dement (Amst).

[15] Hemingway H, Marmot M (1999). Clinical evidence: psychosocial factors in the etiology and prognosis of coronary heart disease: systematic review of prospective cohort studies. West J Med.

[16] Wulsin LR, Singal BM (2003). Do depressive symptoms increase the risk for the onset of coronary disease? A systematic quantitative review. Psychosom Med.

[17] World Health Organization (2019). Risk Reduction of Cognitive Decline and Dementia: WHO Guidelines.

[18] Goldstein MG, Whitlock EP, DePue J, Planning Committee of the Addressing Multiple Behavioral Risk Factors in Primary Care Project (2004). Multiple behavioral risk factor interventions in primary care. Summary of research evidence. Am J Prev Med.

[19] Grunfeld E, Manca D, Moineddin R, Thorpe KE, Hoch JS, Campbell-Scherer D, Meaney C, Rogers J, Beca J, Krueger P, Mamdani M, BETTER Trial Investigators (2013). Improving chronic disease prevention and screening in primary care: results of the BETTER pragmatic cluster randomized controlled trial. BMC Fam Pract.

[20] Middleton KR, Anton SD, Perri MG (2013). Long-term adherence to health behavior change. Am J Lifestyle Med.

[21] Locke EA (1996). Motivation through conscious goal setting. Appl Prev Psychol.

[22] The Department of Health (2013). National Primary Health Care Strategic Framework.

[23] Shelley DR, Gepts T, Siman N, Nguyen AM, Cleland C, Cuthel AM, Rogers ES, Ogedegbe O, Pham-Singer H, Wu W, Berry CA (2020). Cardiovascular disease guideline adherence: an RCT using practice facilitation. Am J Prev Med.

[24] van Charante EP, Richard E, Eurelings LS, van Dalen J, Ligthart SA, van Bussel EF, Hoevenaar-Blom MP, Vermeulen M, van Gool WA (2016). Effectiveness of a 6-year multidomain vascular care intervention to prevent dementia (preDIVA): a cluster-randomised controlled trial. Lancet.

[25] Kim S, McMaster M, Torres S, Cox KL, Lautenschlager N, Rebok GW, Pond D, D'Este C, McRae I, Cherbuin N, Anstey KJ (2018). Protocol for a pragmatic randomised controlled trial of body brain life-general practice and a lifestyle modification programme to decrease dementia risk exposure in a primary care setting. BMJ Open.

[26] Fairweather-Schmidt AK, Anstey KJ (2012). Prevalence of suicidal behaviours in two Australian general population surveys: methodological considerations when comparing across studies. Soc Psychiatry Psychiatr Epidemiol.

[27] Department of Health (2019). GP bulk-billing rates by electorate. 2019-2020 Supplementary Budget Estimates.

[28] Folstein MF, Folstein SE, McHugh PR (1975). 'Mini-mental state'. A practical method for grading the cognitive state of patients for the clinician. J Psychiatr Res.

[29] Sealed Envelope.

[30] Kim S, Cherbuin N, Anstey KJ (2016). Assessing reliability of short and tick box forms of the ANU-ADRI: convenient alternatives of a self-report Alzheimer's disease risk assessment. Alzheimers Dement (N Y).

[31] Anstey KJ, Cherbuin N, Herath PM (2013). Development of a new method for assessing global risk of Alzheimer's disease for use in population health approaches to prevention. Prev Sci.

[32] Anstey KJ, Cherbuin N, Herath PM, Qiu C, Kuller LH, Lopez OL, Wilson RS, Fratiglioni L (2014). A self-report risk index to predict occurrence of dementia in three independent cohorts of older adults: the ANU-ADRI. PLoS One.

[33] Andrews SJ, Eramudugolla R, Velez JI, Cherbuin N, Easteal S, Anstey KJ (2017). Validating the role of the Australian national university Alzheimer's disease risk index (ANU-ADRI) and a genetic risk score in progression to cognitive impairment in a population-based cohort of older adults followed for 12 years. Alzheimers Res Ther.

[34] Craig CL, Marshall AL, Sjöström M, Bauman AE, Booth ML, Ainsworth BE, Pratt M, Ekelund U, Yngve A, Sallis JF, Oja P (2003). International physical activity questionnaire: 12-country reliability and validity. Med Sci Sports Exerc.

[35] Buysse DJ, Reynolds CF, Monk TH, Berman SR, Kupfer DJ (1989). The Pittsburgh sleep quality index: a new instrument for psychiatric practice and research. Psychiatry Res.

[36] Ware J, Kosinski M, Keller SD (1996). A 12-item short-form health survey: construction of scales and preliminary tests of reliability and validity. Med Care.

[37] Collins CE, Burrows TL, Rollo ME, Boggess MM, Watson JF, Guest M, Duncanson K, Pezdirc K, Hutchesson MJ (2015). The comparative validity and reproducibility of a diet quality index for adults: the Australian recommended food score. Nutrients.

[38] Pincus T, Swearingen C, Wolfe F (1999). Toward a multidimensional health assessment questionnaire (MDHAQ): assessment of advanced activities of daily living and psychological status in the patient-friendly health assessment questionnaire format. Arthritis Rheum.

[39] Norton K (2012). New Australian Standard for Adult Pre-Exercise Screening. Informit.

[40] Reitan RM (1971). Trail making test results for normal and brain-damaged children. Percept Mot Skills.

[41] Smith A (1973). (SDMT) Symbol Digit Modalities Test. Western Psychological Services.

[42] Anstey KJ, Bahar-Fuchs A, Herath P, Rebok GW, Cherbuin N (2013). A 12-week multidomain intervention versus active control to reduce risk of Alzheimer's disease: study protocol for a randomized controlled trial. Trials.

[43] White IR, Horton NJ, Carpenter J, Pocock SJ (2011). Strategy for intention to treat analysis in randomised trials with missing outcome data. Br Med J.

[44] Rubin D (2004). Multiple Imputation for Nonresponse in Surveys.

[45] Kivipelto M, Mangialasche F, Ngandu T (2018). Lifestyle interventions to prevent cognitive impairment, dementia and Alzheimer disease. Nat Rev Neurol.

[46] Cherbuin N, Shaw ME, Walsh E, Sachdev P, Anstey KJ (2019). Validated Alzheimer's disease risk index (ANU-ADRI) is associated with smaller volumes in the default mode network in the early 60s. Brain Imaging Behav.

[47] Anstey KJ, Bahar-Fuchs A, Herath P, Kim S, Burns R, Rebok GW, Cherbuin N (2015). Body brain life: a randomized controlled trial of an online dementia risk reduction intervention in middle-aged adults at risk of Alzheimer's disease. Alzheimers Dement (N Y).

