# Supplementary material for: An Internet-Based Intervention Augmented With a Diet and Physical Activity Consultation to Decrease the Risk of Dementia in At-Risk Adults in a Primary Care Setting: Pragmatic Randomized Controlled Trial
Source: J Med Internet Res. 2020 Sep 24;22(9):e19431. doi: 10.2196/19431 (PMC7545332; doi:10.2196/19431)
Supplement: Multimedia Appendix 2 [file jmir_v22i9e19431_app2.docx]

Multimedia Appendix 2. Baseline characteristics by completion^a^

|  | Did not complete | Completed | Test statistic^b^ |
| --- | --- | --- | --- |

| Variable | N | Mean^c^ | *SD^c^* | N | Mean^c^ | *SD^c^* | *t* | *df* | *P* value |
| --- | --- | --- | --- | --- | --- | --- | --- | --- | --- |
| Female | 76 | 54 | 71% | 49 | 32 | 65% | .68 |  | .498 |
| Age (years) | 76 | 49.42 | 13.35 | 49 | 53.02 | 13.03 | -1.49 | 123 | .14 |
| Education (years) | 76 | 16.03 | 4.52 | 49 | 15.97 | 3.93 | .08 | 123 | .936 |
| BMI (kg/m^2^) | 76 | 34.11 | 6.60 | 49 | 33.8 | 7.73 | .24 | 123 | .808 |
| ANU-ADRI-SF^d^ | 67 | 3.24 | 6.65 | 44 | 1.8 | 7.22 | 1.08 | 109 | .282 |
| Cognition z-score | 71 | -0.1 | 0.92 | 48 | 0.15 | 1.11 | -1.31 | 117 | .192 |
| Total MVPA per wk^e^ | 68 | 926.06 | 504.50 | 45 | 955.13 | 391.45 | -.33 | 111 | .744 |
| Sufficient PA^f^ | 67 | 32 | 48% | 44 | 25 | 57% | -.93 |  | .35 |
| CES-D score^g^ | 76 | 9 | 4,15 | 49 | 7 | 3,15 | .74 |  | .461 |
| Diet (ARFS)^h^ | 72 | 35.07 | 9.31 | 48 | 37.21 | 8.82 | -1.26 | 118 | .211 |
| Sleep (PSQI)^i^ | 74 | 7.35 | 4.15 | 49 | 7.41 | 3.82 | -.08 | 121 | .939 |
| SF-12 PCS^j^ | 74 | 44.54 | 8.67 | 49 | 47.24 | 9.40 | -1.63 | 121 | .105 |
| SF-12 MCS^k^ | 74 | 43.28 | 11.47 | 49 | 46.01 | 11.01 | -1.31 | 121 | .192 |
| Diabetes risk (Ausdrisk) | 70 | 15.89 | 5.34 | 48 | 17.04 | 6.63 | -1.05 | 116 | .298 |
| Framingham CVD Risk score^l^ | 61 | 3 | 0,6 | 46 | 4.5 | 1,8 | -1.51 |  | .13 |
| Intervention Group; n, %  BBL-GP^m^  LMP^n^  Control |  | 29  24  23 | 69%  59%  55% |  | 13  17  19 | 31%  41%  45% | 1.92 | 2 | 0.381 |
| ^a^Completion defined as undertaking all five assessments.  ^b^Test statistic Z test for difference between proportions for sex and sufficient physical activity, and rank sum test for CES-D and Framingham CVD risk score; t-test for all other variables.  ^c^N, % presented for sex and sufficient physical activity. Median, Q1, Q3 presented for CES-D and Framingham CVD risk score.  ^d^ANU-ADRI-SF: ANU-Alzheimer’s Disease Risk Index Short-Form.  ^e^MVPA – PA: Total minutes of Moderate-Vigorous Physical Activity per week (activity registering 3 or more metabolic equivalents for at least 10 minutes).  ^f^PA: Physical activity.  ^g^CES-D: Centre for Epidemiological Studies Depression Scale.  ^h^ARFS: Australian Recommended Food Score.  ^i^PSQI: Pittsburgh Sleep Quality Index.  ^j^SF-12-PCS: Short-Form Health Survey Physical Component Score.  ^k^SF-12 MCS: Short-Form Health Survey Mental Component Score.  ^l^CVD: Cardiovascular disease.  ^m^BBL-GP: Body, Brain, Life-General Practice.  ^n^LMP: Lifestyle Modification Programme. | | | | | | | | | |
