# Supplementary material for: An Internet-Based Intervention Augmented With a Diet and Physical Activity Consultation to Decrease the Risk of Dementia in At-Risk Adults in a Primary Care Setting: Pragmatic Randomized Controlled Trial
Source: J Med Internet Res. 2020 Sep 24;22(9):e19431. doi: 10.2196/19431 (PMC7545332; doi:10.2196/19431)
Supplement: Multimedia Appendix 3 [file jmir_v22i9e19431_app3.docx]

Multimedia Appendix 3. Difference in outcomes between groups at each follow-up

| Outcome | Follow-up Time | BBL-GP^a^ vs Control | | | | LMP^b^ vs Control | | | | BBL-GP^a^ vs LMP^b^ | | | | Interaction test | | |
| --- | --- | --- | --- | --- | --- | --- | --- | --- | --- | --- | --- | --- | --- | --- | --- | --- |
|  |  | Estimate^c^ | Lower CI | Upper CI | *P* value | Estimate^c^ | Lower CI | Upper CI | *P* value | Estimate^c^ | Lower CI | Upper CI | *P* value | Chi-sq | *df* | *P*  value |
|  |  |  |  |  |  |  |  |  |  |  |  |  |  |  |  |  |
| **ANU-ADRI-SF^d^** | Immediate | -2.30 | *-5.93* | *1.34* | 0.220 | -0.49 | *-3.86* | *2.88* | 0.780 | -1.81 | *-5.37* | *1.76* | 0.320 |  |  |  |
|  | Week 18 | -2.49 | -5.99 | 1.02 | 0.160 | -0.20 | -3.60 | 3.19 | 0.910 | -2.28 | -5.87 | 1.31 | 0.210 |  |  |  |
|  | Week 36 | -3.43 | -7.16 | 0.29 | 0.070 | -0.79 | -4.18 | 2.59 | 0.650 | -2.64 | -6.31 | 1.03 | 0.160 |  |  |  |
|  | Week 62 | -3.06 | -6.71 | 0.60 | 0.100 | -1.24 | -4.82 | 2.34 | 0.500 | -1.82 | -5.53 | 1.90 | 0.340 | 17.51 | 8.00 | 0.030 |
| **Standardised cognition score** | Week 18 | -0.18 | -0.54 | 0.19 | 0.350 | -0.12 | -0.49 | 0.24 | 0.510 | -0.05 | -0.43 | 0.32 | 0.780 |  |  |  |
|  | Week 36 | -0.16 | -0.56 | 0.23 | 0.420 | 0.06 | -0.31 | 0.43 | 0.740 | -0.23 | -0.62 | 0.17 | 0.270 |  |  |  |
|  | Week 62 | -0.17 | -0.56 | 0.23 | 0.410 | 0.04 | -0.35 | 0.43 | 0.820 | -0.21 | -0.61 | 0.19 | 0.300 | 2.94 | 6.00 | 0.820 |
| **Total MVPA per week^e^** | Week 18 | 223.69 | -6.19 | 453.56 | 0.060 | -92.28 | -312.9 | 128.36 | 0.410 | 315.97 | 81.87 | 550.07 | 0.010 |  |  |  |
|  | Week 36 | -5.34 | -254.2 | 243.52 | 0.970 | -30.80 | -268.3 | 206.68 | 0.800 | 25.47 | -220.5 | 271.42 | 0.840 | 7.82 | 4.00 | 0.100 |
| **Sufficient PA^f^** | Immediate | 14.56 | 0.39 | 549.11 | 0.150 | 0.23 | 0.03 | 2.06 | 0.190 | 63.85 | 1.81 | 2248.65 | 0.020 |  |  |  |
|  | Week 18 | 1.77 | 0.17 | 18.17 | 0.630 | 2.23 | 0.27 | 18.65 | 0.460 | 0.79 | 0.07 | 8.81 | 0.850 |  |  |  |
|  | Week 36 | 2.34 | 0.11 | 49.99 | 0.590 | 0.70 | 0.08 | 6.27 | 0.750 | 3.36 | 0.17 | 64.89 | 0.420 |  |  |  |
|  | Week 62 | 0.61 | 0.04 | 9.01 | 0.720 | 0.82 | 0.07 | 9.50 | 0.870 | 0.74 | 0.05 | 10.59 | 0.830 | 7.44 | 8.00 | 0.490 |
| **CES-D^g^** | Immediate | 0.89 | 0.56 | 1.40 | 0.610 | 1.03 | 0.68 | 1.57 | 0.880 | 0.86 | 0.55 | 1.35 | 0.510 |  |  |  |
|  | Week 18 | 1.24 | 0.81 | 1.90 | 0.320 | 1.01 | 0.67 | 1.53 | 0.970 | 1.23 | 0.80 | 1.89 | 0.350 |  |  |  |
|  | Week 36 | 1.02 | 0.65 | 1.59 | 0.950 | 0.89 | 0.59 | 1.36 | 0.600 | 1.14 | 0.73 | 1.77 | 0.570 |  |  |  |
|  | Week 62 | 1.03 | 0.65 | 1.63 | 0.900 | 1.01 | 0.65 | 1.57 | 0.960 | 1.02 | 0.65 | 1.61 | 0.940 | 5.94 | 8.00 | 0.650 |
| **Diet (ARFS)^h^** | Immediate | 3.36 | -1.99 | 8.70 | 0.220 | 1.56 | -3.21 | 6.32 | 0.520 | 1.80 | -3.53 | 7.12 | 0.510 |  |  |  |
|  | Week 18 | 2.05 | -3.06 | 7.16 | 0.430 | -3.30 | -8.20 | 1.61 | 0.190 | 5.35 | 0.28 | 10.42 | 0.040 |  |  |  |
|  | Week 36 | 4.64 | -0.68 | 9.95 | 0.090 | 0.57 | -4.28 | 5.41 | 0.820 | 4.07 | -1.20 | 9.34 | 0.130 |  |  |  |
|  | Week 62 | 2.29 | -3.12 | 7.71 | 0.410 | -1.22 | -6.33 | 3.88 | 0.640 | 3.52 | -1.90 | 8.94 | 0.200 | 11.94 | 8.00 | 0.150 |
| **Sleep (PSQI)^i^** | Immediate | -2.04 | -3.99 | -0.09 | 0.040 | -0.87 | -2.70 | 0.96 | 0.350 | -1.17 | -3.11 | 0.77 | 0.240 |  |  |  |
|  | Week 18 | -0.63 | -2.50 | 1.25 | 0.510 | -0.03 | -1.87 | 1.81 | 0.980 | -0.60 | -2.50 | 1.30 | 0.540 |  |  |  |
|  | Week 36 | -0.73 | -2.71 | 1.26 | 0.470 | -1.97 | -3.83 | -0.10 | 0.040 | 1.24 | -0.72 | 3.20 | 0.220 |  |  |  |
|  | Week 62 | -0.05 | -2.09 | 1.98 | 0.960 | -0.48 | -2.41 | 1.45 | 0.620 | 0.43 | -1.62 | 2.48 | 0.680 | 16.83 | 8.00 | 0.030 |
| ^a^BBL-GP: Body, Brain, Life-General Practice.  ^b^LMP: Lifestyle Modification Programme.  ^c^Difference in means presented for all outcomes except Sufficient PA for which the measure of effect is the odds ratio; Results from regression models adjusted for sex and age.  ^d^ANU-ADRI-SF: ANU-Alzheimer’s Disease Risk Index Short-Form.  ^e^MVPA – PA: Total minutes of Moderate-Vigorous Physical Activity per week (activity registering 3 or more metabolic equivalents for at least 10 minutes).  ^f^PA: Physical activity.  ^g^CES-D: Centre for Epidemiological Studies Depression Scale.  ^h^ARFS: Australian Recommended Food Score.  ^i^PSQI: Pittsburgh Sleep Quality Index. | | | | | | | | | | | | | | | | |
