# Supplementary material for: An Internet-Based Intervention Augmented With a Diet and Physical Activity Consultation to Decrease the Risk of Dementia in At-Risk Adults in a Primary Care Setting: Pragmatic Randomized Controlled Trial
Source: J Med Internet Res. 2020 Sep 24;22(9):e19431. doi: 10.2196/19431 (PMC7545332; doi:10.2196/19431)
Supplement: Multimedia Appendix 4 [file jmir_v22i9e19431_app4.docx]

Multimedia Appendix 4. Difference in outcomes between groups at each follow-up, adjusted for baseline differences

| Outcome | Follow-up Time | BBL-GP^a^ vs Control | | | | LMP^b^ vs Control | | | | BBL-GP^a^ vs LMP^b^ | | | | Interaction test | | |
| --- | --- | --- | --- | --- | --- | --- | --- | --- | --- | --- | --- | --- | --- | --- | --- | --- |
|  |  | Estimate^c^ | Lower CI | Upper CI | *P* value | Estimate^c^ | Lower CI | Upper CI | *P* value | Estimate^c^ | Lower CI | Upper CI | *P* value | Chi-sq | *df* | *P*  value |
| **ANU-ADRI-SF^d^** | Immediate | -3.86 | -6.81 | -0.90 | 0.010 | -1.78 | -4.44 | 0.87 | 0.190 | -2.08 | -4.94 | 0.79 | 0.160 |  |  |  |
|  | Week 18 | -4.05 | -6.81 | -1.28 | <0.001 | -1.50 | -4.15 | 1.16 | 0.270 | -2.55 | -5.44 | 0.34 | 0.080 |  |  |  |
|  | Week 36 | -4.99 | -8.04 | -1.94 | <0.001 | -2.08 | -4.73 | 0.56 | 0.120 | -2.91 | -5.90 | 0.08 | 0.060 |  |  |  |
|  | Week 62 | -4.62 | -7.62 | -1.62 | <0.001 | -2.53 | -5.43 | 0.37 | 0.090 | -2.09 | -5.18 | 1.01 | 0.190 | 17.51 | 8.00 | 0.030 |
| **Standardised cognition score** | Week 18 | 0.02 | -0.23 | 0.27 | 0.900 | -0.08 | -0.31 | 0.16 | 0.510 | 0.10 | -0.16 | 0.35 | 0.460 |  |  |  |
|  | Week 36 | 0.03 | -0.26 | 0.32 | 0.850 | 0.11 | -0.13 | 0.35 | 0.390 | -0.08 | -0.36 | 0.21 | 0.600 |  |  |  |
|  | Week 62 | 0.03 | -0.26 | 0.31 | 0.860 | 0.09 | -0.18 | 0.36 | 0.520 | -0.06 | -0.35 | 0.22 | 0.670 | 2.94 | 6.00 | 0.820 |
| **Total MVPA^e^ per week** | Week 18 | 86.47 | -96.00 | 268.94 | 0.350 | -33.34 | -201.2 | 134.52 | 0.700 | 119.81 | -67.79 | 307.41 | 0.210 |  |  |  |
|  | Week 36 | -142.55 | -349.5 | 64.38 | 0.180 | 28.14 | -160.0 | 216.31 | 0.770 | -170.69 | -371.8 | 30.39 | 0.100 | 7.82 | 4.00 | 0.100 |
| **Sufficient PA^f^** | Immediate | 17.12 | 0.41 | 710.01 | 0.140 | 0.36 | 0.04 | 3.58 | 0.380 | 47.81 | 1.28 | 1784.47 | 0.040 |  |  |  |
|  | Week 18 | 2.08 | 0.19 | 23.12 | 0.550 | 3.51 | 0.38 | 32.76 | 0.270 | 0.59 | 0.05 | 7.14 | 0.680 |  |  |  |
|  | Week 36 | 2.75 | 0.12 | 63.64 | 0.530 | 1.09 | 0.11 | 10.95 | 0.940 | 2.52 | 0.12 | 51.89 | 0.550 |  |  |  |
|  | Week 62 | 0.72 | 0.04 | 11.75 | 0.810 | 1.29 | 0.10 | 16.43 | 0.850 | 0.56 | 0.03 | 8.93 | 0.680 | 7.44 | 8.00 | 0.490 |
| **CES-D^g^** | Immediate | 0.90 | 0.64 | 1.27 | 0.550 | 1.13 | 0.85 | 1.51 | 0.400 | 0.80 | 0.57 | 1.11 | 0.180 |  |  |  |
|  | Week 18 | 1.26 | 0.93 | 1.70 | 0.140 | 1.10 | 0.83 | 1.47 | 0.490 | 1.14 | 0.84 | 1.55 | 0.410 |  |  |  |
|  | Week 36 | 1.03 | 0.74 | 1.44 | 0.870 | 0.98 | 0.73 | 1.31 | 0.880 | 1.05 | 0.76 | 1.46 | 0.760 |  |  |  |
|  | Week 62 | 1.04 | 0.73 | 1.48 | 0.820 | 1.11 | 0.81 | 1.52 | 0.530 | 0.94 | 0.67 | 1.33 | 0.730 | 5.94 | 8.00 | 0.650 |
| **Diet (ARFS) ^h^** | Immediate | 3.63 | -1.03 | 8.29 | 0.130 | 1.68 | -2.28 | 5.65 | 0.400 | 1.94 | -2.66 | 6.54 | 0.410 |  |  |  |
|  | Week 18 | 2.33 | -2.05 | 6.70 | 0.300 | -3.17 | -7.28 | 0.94 | 0.130 | 5.50 | 1.20 | 9.80 | 0.010 |  |  |  |
|  | Week 36 | 4.91 | 0.30 | 9.52 | 0.040 | 0.69 | -3.35 | 4.73 | 0.740 | 4.22 | -0.32 | 8.75 | 0.070 |  |  |  |
|  | Week 62 | 2.57 | -2.15 | 7.29 | 0.290 | -1.10 | -5.44 | 3.25 | 0.620 | 3.66 | -1.04 | 8.37 | 0.130 | 11.94 | 8.00 | 0.150 |
| **Sleep (PSQI) ^i^** | Immediate | -0.96 | -2.46 | 0.55 | 0.210 | -0.60 | -1.96 | 0.76 | 0.390 | -0.35 | -1.84 | 1.14 | 0.640 |  |  |  |
|  | Week 18 | 0.46 | -0.94 | 1.86 | 0.520 | 0.24 | -1.13 | 1.60 | 0.730 | 0.22 | -1.22 | 1.66 | 0.760 |  |  |  |
|  | Week 36 | 0.36 | -1.19 | 1.91 | 0.650 | -1.70 | -3.09 | -0.30 | 0.020 | 2.06 | 0.54 | 3.58 | 0.010 |  |  |  |
|  | Week 62 | 1.03 | -0.59 | 2.65 | 0.210 | -0.22 | -1.70 | 1.27 | 0.780 | 1.25 | -0.38 | 2.88 | 0.130 | 16.83 | 8.00 | 0.030 |
| ^a^BBL-GP: Body, Brain, Life-General Practice.  ^b^LMP: Lifestyle Modification Programme.  ^c^Difference in means presented for all outcomes except Sufficient PA for which the measure of effect is the odds ratio; Results from regression models adjusted for sex and age; Estimates adjusted for baseline differences in outcomes.  ^d^ANU-ADRI-SF: ANU-Alzheimer’s Disease Risk Index Short-Form.  ^e^MVPA – PA: Total minutes of Moderate-Vigorous Physical Activity per week (activity registering 3 or more metabolic equivalents for at least 10 minutes).  ^f^PA: Physical activity.  ^g^CES-D: Centre for Epidemiological Studies Depression Scale.  ^h^ARFS: Australian Recommended Food Score.  ^i^PSQI: Pittsburgh Sleep Quality Index. | | | | | | | | | | | | | | | | |
