# Supplementary material for: An Internet-Based Intervention Augmented With a Diet and Physical Activity Consultation to Decrease the Risk of Dementia in At-Risk Adults in a Primary Care Setting: Pragmatic Randomized Controlled Trial
Source: J Med Internet Res. 2020 Sep 24;22(9):e19431. doi: 10.2196/19431 (PMC7545332; doi:10.2196/19431)
Supplement: Multimedia Appendix 5 [file jmir_v22i9e19431_app5.docx]

Multimedia Appendix 5. Summary of participant feedback

| Feedback | | Rating | |
| --- | --- | --- | --- |
|  |  | N | % |
| **Overall experience of the study** | |  |  |
|  | Very bad | 0 | 0.0 |
|  | Bad | 0 | 0.0 |
|  | Fair | 8 | 27.6 |
|  | Good | 20 | 69.0 |
|  | Very good | 1 | 3.4 |
| **Whether participation was worthwhile** | |  |  |
|  | Yes – learned a lot and felt it was beneficial for me | 12 | 46.2 |
|  | Yes, but there were some aspects I did not enjoy | 8 | 30.8 |
|  | No, but I found some aspects interesting and informative | 5 | 19.2 |
|  | No – I did not get any benefit from participating | 1 | 3.8 |
| **Relevance of material** | |  |  |
|  | Not at all relevant | 0 | 0.0 |
|  | Slightly relevant | 1 | 3.5 |
|  | Moderately relevant | 9 | 32.1 |
|  | Mostly relevant | 14 | 50.0 |
|  | Very relevant | 4 | 14.3 |
| **Interest of material** | |  |  |
|  | Not at all interesting | 0 | 0.0 |
|  | Slightly interesting | 2 | 7.4 |
|  | Moderately interesting | 8 | 29.6 |
|  | Mostly interesting | 12 | 44.4 |
|  | Very interesting | 5 | 18.5 |
| **Repetition of material** | |  |  |
|  | Not very repetitive | 2 | 7.1 |
|  | Somewhat repetitive | 4 | 14.3 |
|  | Fair amount of repetition | 17 | 60.7 |
|  | Mostly repetitive | 5 | 17.9 |
|  | Very repetitive | 0 | 0.0 |
| **Perception of effectiveness of randomised group** | |  |  |
|  | Most effective treatment | 5 | 17.2 |
|  | Least effective treatment | 12 | 41.4 |
|  | Don’t know | 12 | 41.4 |
